# Religion and Fertility in Contemporary Northern Ireland

**DOI:** 10.1007/s10680-016-9399-8

**Published:** 2016-09-28

**Authors:** Patrick McGregor, Patricia McKee

**Affiliations:** 1 18 Piney Lane, Belfast, BT9 5QS UK; 2 University of Ulster, Shore Road, Belfast, BT37 0QB UK

**Keywords:** Fertility analysis, Protestant and Catholic fertility in Northern Ireland, Religion and fertility, Fertility by religious affiliation and none

## Abstract

Northern Ireland has been and continues to be deeply divided on the basis of religion. This paper examines and compares contemporary fertility in the two communities given the sharp declines that have occurred in recent decades. The data are drawn from the Northern Ireland Longitudinal Study and cover 108,000 women aged 16–44 in the period 1997–2007. A logit analysis of births in the period is undertaken based on individual demographic data and also the characteristics of the locality in which the woman is resident. The effect of religion is measured by its individual marginal effect averaged over the total sample, over time and by the age of the woman. The estimated average marginal effect is 4 % of the probability of a woman having a birth over the entire period. The effect peaks when the woman is between 29 and 30 years and is stable over time. When the fertility behaviour of each religious group is compared separately with those women that came from the same background but had lost their religion, it is found to differ substantially. Thus, community background is rejected as a possible explanation of the difference in fertility between declared Catholics and Protestants. Although the fertility rates of former Catholics and Protestants appear to be converging, the size of these groups is relatively small. There is no evidence to suggest that the small but distinct difference in the fertility rates of the two religious communities is likely to change in the immediate future.

**Electronic supplementary material:**

The online version of this article (doi:10.1007/s10680-016-9399-8) contains supplementary material, which is available to authorized users.

## Introduction and Background

The state of Northern Ireland was formed in 1921 and comprised six of the thirty-two counties of Ireland. The division of the island was an attempt to accommodate violently contradictory unionist and nationalist aspirations. In the South of Ireland, the abandonment of the six northern counties precipitated a civil war in which the Republican forces were defeated. In the North, a Protestant state was formed excluding a substantial Catholic minority. In 1926, when the first Census of Northern Ireland was undertaken, one-third of the population of one and a quarter million was Catholic and overwhelmingly nationalist. Politically, they were committed to a single state on the island of Ireland (see Bardon 2001 for a full historical overview).

Northern Ireland became an archetype of a divided society, with a Catholic minority discriminated against in terms of public housing, local government representation and employment and a Protestant majority, fearful of the erosion of any of its privileges. The struggle for political rights for the Catholic minority developed in the late 1960s but degenerated into violent confrontation. The next 40 years became characterized as ‘The Troubles’ in which over three thousand people died.

Fertility, within a political perspective, was of interest because of the consequences that differential community fertility rates could have for the population composition and thus for votes. For example, Compton’s examination of the 1981 Census results was a critical analysis of the proposition that the Protestant majority will inevitably disappear because of the greater demographic vitality of the Roman Catholic population (1985: 201).

In 1983, the Northern Ireland Fertility Study (NIFS) was undertaken and its findings published in a book by Compton and Coward (1989). Northern Ireland was, according to these authors, subject to two overlapping demographic regimes—the pre-transition Irish one with high marital fertility moderated by less than universal marriage and the British regime with low, planned fertility accompanied by comparatively universal marriage. Within Northern Ireland the ‘majority Protestant population … exhibits all the characteristic features of the British regime, while the demographic behaviour of the Catholic population places it firmly within the Irish regime’ (Compton and Coward 1989: 19).

This paper seeks to determine the contribution of religion to fertility in contemporary Northern Ireland, based on self-designated religion in the 2001 Census. The different fertility regimes associated with self-defined Protestants and Catholics are of central interest. In the 2001 Census, 40.3 % of the population stated their religion to be Catholic versus 45.6 % Protestant and 13.9 % either had no religion or declined to state it. In this last group, 43.8 % have a Catholic background (referred to as former Catholics further) and 53.1 % are former Protestants (NISRA 2002). While the difference in fertility rates between Protestants and Catholics has dominated the political discourse, it is crucial to investigate the differences between Protestants and former Protestants (and similarly for Catholics) to understand the specific role of religion.

**Fig. 1:**
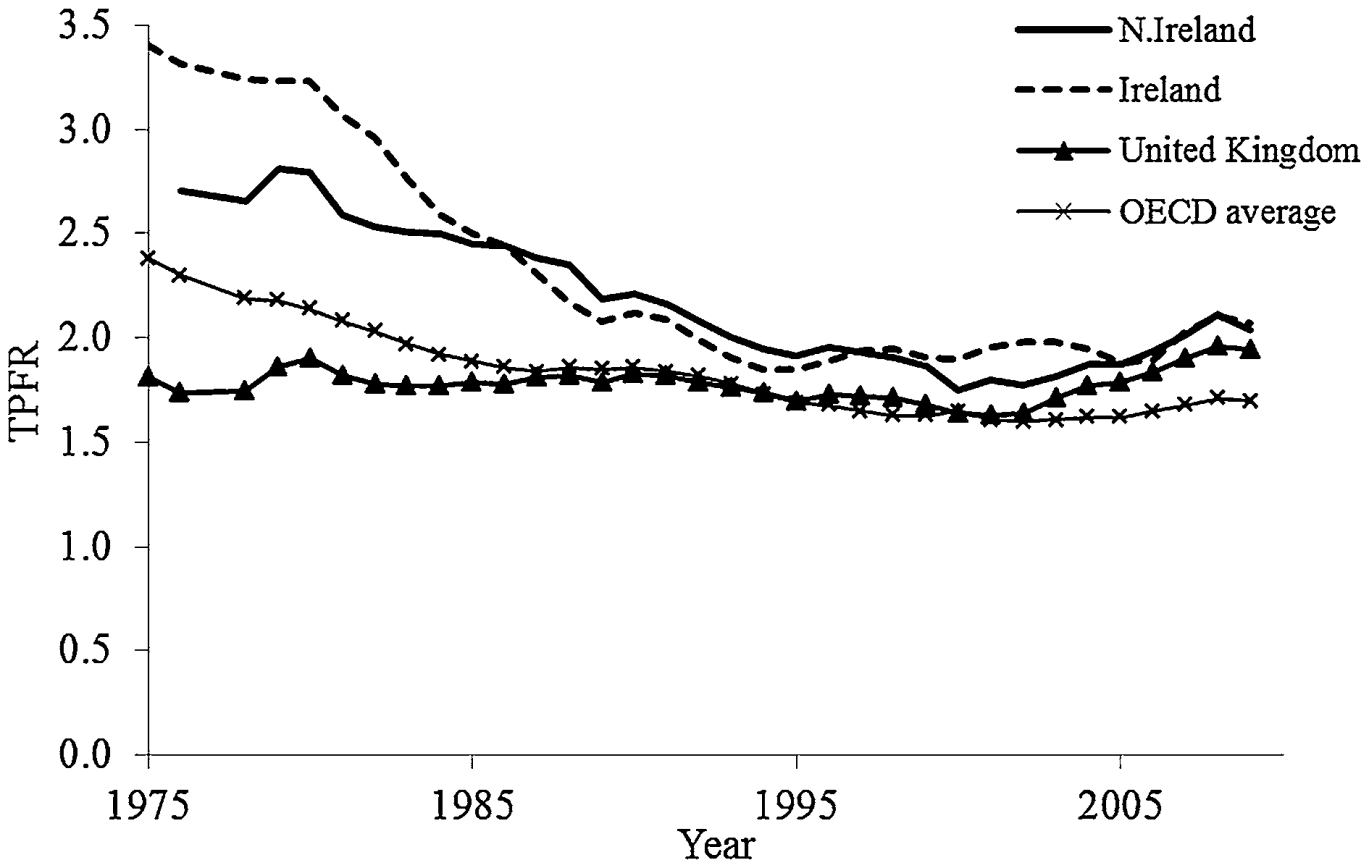
Total period fertility rates in selected countries. *Sources*: NISRA (2013b), OECD (2011)

In the three decades since the NIFS was undertaken, union and family formation have changed. For example, the role of marriage considerably lost importance. In 2010, 40 % of births in Northern Ireland occurred outside marriage compared to 9 % in 1983. Over a similar period, the total period fertility rate (TPFR) decreased from 2.79 in 1980 to 1.75 in 2000 though in 2008 it rebounded to 2.05 (NISRA 2013a, b: Chart 3). As is evident from Fig. 1, the 1980s witnessed a dramatic decline in the TPFR in the Republic as well. While Irish fertility is still above the OECD average rate, the trends it exhibits are similar to Northern Ireland and indeed the rest of Europe.

The trends exhibited in Fig. 1 have been explained within neoclassical economics by Becker (1991). As a couple’s income increased, it would be anticipated that they would consume more of the services, such as parental feeling and company, generated by children. These services depend both on the number of children a couple has but also on the resources they allocate to the development of each child, which determines the ‘quality’ of the child. Becker’s model can explain the fall of fertility despite an increase in income since child services can increase even though the number of children borne falls, provided the elasticity with respect to child quality dominates the negative elasticity with respect to their number (see Hotz et al. 1997).

Becker’s model could also explain differential fertility due to religion if this operated through either income or utility. In the first case, this could operate in the area of discrimination either directly or through differential access to higher education, an important source of human capital. There is no evidence of this (Osborne and Gallagher 2003); For the past three decades Northern Ireland has been subject to strict equality legislation which has reduced the effects of the systematic discrimination inherited from the past (McLaughlin 2007).

Secondly, if religious belief moulds tastes and preferences, then it is possible that Catholics could weigh quantity higher than quality compared to Protestants and thus have a higher relative fertility. Such preferences may be due to doctrinal differences. Certainly, religion continues to play an important role in Northern Ireland. In 1998, 10 % (13 %) of Catholics (Protestants) classed themselves as either ‘extremely’ or ‘very religious’ according to the Life and Times Survey (NILTS). Equivalent figures exist for 2008 (12 vs. 17 %). Those who considered themselves ‘somewhat religious’ grew from 56 % (42 %) to 60 % (44 %) in the same period (NILTS 1998, 2008).

In the mainstream Protestant religions, fertility is considered a matter of individual choice (Lehrer 1996). In contrast, the doctrine of the Roman Catholic Church is pronatalist. This suggests that Catholics would rank quantity more highly than quality compared to Protestants. However, there has been a substantial decline in Catholic fertility despite extremely limited doctrinal change; Italy has been classed as having ‘lowest-low fertility’ by Kohler et al. (2002); in the USA Catholic and non-Catholic fertility was virtually identical by the 1970s (Frejka and Westoff 2006). Doctrinal differences are substantial but appear to have limited effects on fertility in many cases.

This is true in Ireland as well as internationally. The term ‘à la carte’ Catholic has been coined by one local observer of Catholicism in Ireland (Inglis 2009 also see 1998) to describe a person who attends Mass regularly but distances themselves from the Church’s teaching on contraception, sexual intercourse outside marriage, divorce, etc., which are considered matters of individual conscience. Personal beliefs of some Catholics and Protestants would be hard to distinguish from each other.

The use of self-designation in ascribing religious affiliation means that differences in fertility between religious communities cannot be definitely attributed to doctrinal differences. In Northern Ireland it is possible that any such differences, particularly if they are small, may be due to separation. Residential segregation in Northern Ireland has been marked and stable for over four decades (Shuttleworth and Lloyd 2009), and this has been reinforced within the education system. In 2010–2011, 97 % (89 %) of Catholic pupils in the primary (secondary) sector are in Catholic-managed schools (DENI 2012). Establishing the relative importance of doctrine and separation in fertility is a further issue discussed by this paper.

While comparing the fertility of both communities with those who currently have none but were brought up with one allows the (pure) effect of religion to be established; comparing those who have lost the Protestant or Catholic religion of their upbringing gives an important measure of the extent of secularization in Northern Ireland. Across Western Europe this has been associated with the decline of religion (Surkyn and Lesthaeghe 2004). The extent to which losing religion leads to a homogeneous group, at least with regard to fertility, is an important development for the future of a country which has been blighted in the past by sectarianism.

The research questions of this paper cannot be addressed using current official statistics. The birth registrations do not contain any information on religion, and the Census does not ask how many births a woman has had. To obtain the necessary data, the Northern Ireland Longitudinal Study (NILS) is used. In this study, 2001 and 1991 Census returns and birth registrations can be linked at the individual level. This results in annual fertility data of a panel of over 108,000 women covering the years 1997–2007. The construction of these data, referred to hereafter as the fertility panel, is central for addressing the research questions outlined above.

## Data, Measurement and Analytical Strategy

The Northern Ireland Longitudinal Survey provides the individual data that are used in the construction of the fertility panel. The NILS does not conduct any population surveys itself but rather anonymizes the individual returns used in the construction of the official, aggregate data. The official data on birth registrations and the Censuses are reviewed in the first two subsections which are then followed by a subsection that outlines how these data are combined by the NILS. The final two subsections are devoted to the statistical nature of the study.

### The Birth Registration Data

Birth registrations are a legal obligation and fall under the responsibility of the General Register Office (GRO). The data derived from the registrations (and mid-year population projections based on the Census) are aggregated and summarized in tables that accompany the Annual Report of the Registrar General (see, for example, NISRA 2007). Whereas no information concerning religion is requested at the registration of a birth, the parity of the mother at the time of the new birth is recorded.

### The Census Data

The enumeration form used by the decennial Census contains information at the household and the individual level. The former includes the relationships between all members of the household. Furthermore, questions on the religion of each individual in the household are included. Unfortunately, there is no question concerning the parity of a woman in the 2001 Census. Nevertheless, this can be derived from the number of individuals in the household that refer to the NILS member as ‘mother’. This estimate can be improved by using the 1991 Census and the GRO data (see above and in Data Appendix for full details).

The enumeration form of the Census asks whether the individual belongs to any particular religion. For those respondents answering ‘yes’, religious affiliation can be aggregated into Catholic, Protestant (and other Christian) and Other. In the statistical analysis, Catholics and Protestants are represented by dummy variables. If respondents answered ‘no’, then and only then, the respondent is asked what religion he or she was brought up in (which can be aggregated as mentioned before). This information permits former religion also to be included as dummy variables.

It is important in this work that the Census information on religion is unbiased. In 1981, 18.5 % of respondents declined to state their religion. In 2001, ‘no religion or not stated’ amounted to 13.5 % of the enumerated population. Unfortunately, the two groups were combined during processing of the Census. In an analysis of the raw data, approximately 10 % stated ‘no religion’ and 4 % left the question unanswered (Registrar General Northern Ireland 1993; NISRA 2013a, b). The difference between the response rates in 1981 and 2001 can be related directly to the level of violence. Whereas in 1981, 114 people were killed as a consequence of the Troubles, in 2001 16 were killed (Sutton 2001). Following ceasefires from the Irish Republican Army (IRA) and the Loyalist paramilitaries, the ‘Good Friday Agreement’ was signed in 1998. The agreement was overwhelmingly approved in referenda in Northern Ireland and the Republic of Ireland. Consequently, by 2001 the Troubles had declined to a level that removes anxiety from the answers to the religion questions in the Census of that year.

Individual Census socio-economic information other than religion was not used in the analysis. There were two major reasons for this. Firstly, such information relates to a single year in the panel, whereas socio-economic status is likely to be subject to major change over the period of 11 years. Secondly, given the cross-sectional nature of the data, it is not possible to disentangle decisions related to household formation, education and fertility decisions. Thus, they should be considered endogenous variables (see Öst 2012, as an example).

Published Census data relating to the locality that an individual resided in was employed as an indicator of determinants relevant to individual fertility decisions (such as income) but which are not available at an individual level. The proportion of persons from a Catholic community background, *PERCATH*, is employed as a separate variable in the analysis because of its central role. Eleven socio-economic variables in the 2001 Census are used. Two each relate to qualification, social grade and age, one each to the proportion of houses rented and female economic inactivity and the remaining three are the proportion of households with married or cohabiting or lone parents with dependent children. The locality was taken as the Census Super Output Area (SOA) of which there were 890 in 2001. The rotated principal components (*RPC*) of eleven relevant socio-economic variables from the 2001 Census provided four components, named *RPC1*–*4*, that explain 91 % of the variation in the data. Details can be found  in the online Appendix 1*.

A third of the variance of the Census variables is accounted for by *RPC1* which is the only rotated variable where the Census education variables load substantially. It may thus be considered as inversely related to potential economic well-being. It accounts for a third of the variance in the locality variables. *RPC2* is the only other rotated component that is significant in the regression analysis (see Table 3 below). It is inversely related to social status.

The presence of immigrants from the 2004 Accession countries to the EU (the A8) (see Fegan and Marshall 2008) has been relevant for fertility in Northern Ireland. The focus of this paper is the influence of religion on fertility. Historically, this has been concerned with the native stock (indeed substantial immigration has been a recent development). The use of the 2001 Census ensures though that the fertility sample is made up overwhelmingly of native stock. To capture any influence that immigration might have, *PROA8*_−1_, the proportion of A8 births in the SOA, has been employed in the regression analysis.

### The Northern Ireland Longitudinal Study (NILS)

The NILS consists of a circa 28 % systematic sample of administrative data. The linkage between these databases is based on health card registrations which will cover virtually all the population in Northern Ireland for the concerned year. At this stage the information is limited to date of birth, gender and location. The power of the NILS rests on the linkage of these records with major administrative databases (see O’Reilly et al. 2012) such as the Censuses and the Birth Registration Data used in this case. Projects to utilize the NILS have to meet strict criteria, and the data analysis for approved projects is conducted within a secure environment. The data for each project are constructed specifically for that project by NILS staff and are archived on completion of the project.

The criterion for inclusion in the analysis was that the individual in the NILS was a woman aged 16–44 at any time between 1997 and 2007. Consequently, a panel of women was generated which is further referred to as the fertility panel. If the Birth Registration Data indicated she gave birth in any particular year, a dummy variable was coded to one. Women’s religion was obtained through linking their record to the 2001 Census.

Women’s parity status in 2001 is based upon the relationship matrix in the 2001 Census, revised with data from the 1991 Census and the Birth Registration Data. Her parity for the remaining years in the fertility panel was determined using the Birth Registration Data. The duration a woman had been at her current parity level was determined directly from the annual series of her parity and expressed in years. A detailed account is provided in the online Data Appendix.

### The Statistical Model

In the analysis of aggregate demographic data consisting of event counts of individuals of different ages over different years the age-period-cohort (APC) model is frequently employed (Mason and Wolfinger 2002). Raftery et al. (1995) extended the APC model to individual data and included income, parity and the length of time the woman had experienced her current level of parity (which is referred to as duration below). As income is not available at an individual level in the NILS, locality variables were used. This resulted in an estimation of (1) by a logit model using the variables defined in Table 1. Grouping these variables together allows the propensity of the *i*th woman to have a birth in any particular year, $B^{*}$, to be presented succinctly as a linear function of these sets of variables:

1 $$B_{ij}^{*} = \alpha_{0j} + \alpha_{1j} DEMOG_{ij} + \alpha_{2j} PERIOD_{ij} + \alpha_{3j} COHORT_{ij} + \alpha_{4j} LOCALITY_{ij} + \varepsilon_{ij}$$

where *ɛ* is the random error term and *j* = *CATHOLIC* or *PROTESTANT*. The parameter vectors are allowed, in the first instance, to differ between the two religious groups so *α*_1,*CATHOLIC*_ ≠ *α*_1,*PROTESTANT*_. Now $B^{*}$, the latent index, is not directly observed, and (1) is estimated as a logit.

**Table 1:** The definition of variables

Variable	Definition
*BIRTH*	=1 if woman had a birth in the current year; =0 otherwise
*AGE*	Age of woman in years/30*
*PARi*	Parity of woman at beginning of the current year; =1 if parity = *i*, *i* = 0, 1, 2 and = 0 otherwise
*DUR* > *4*	=1 if duration from previous birth or from 16 is greater than 4 years, =0 otherwise
*DUR04*	Duration in years from previous birth or from 16 if <5 years and *DUR* > 4 = 0
*DUMDUR*	=1 if duration not specific
*PERi*	=1 if year of observations = *i*, *i* = 1998–2007; =0 otherwise
*C x t x*+*4*	=1 if woman is in the cohort born between 19x and 19x+4 where x = 53, 58, 63, 68, 73, 78, 83
*CATHOLIC*	=1 if woman is a Catholic; =0 otherwise
*PROTESTANT*	=1 if woman is a Protestant; =0 otherwise
*f C*	=1 if woman is from a Catholic background and *CATHOLIC* = 0; =0 otherwise
*f P*	=1 if woman is from a Protestant background and *PROTESTANT* = 0; =0 otherwise
*PERCATH*	Proportion of Catholics in the SOA where woman is resident in 2001
*PP*	=(1 − *PERCATH*) if *CATHOLIC* = 0; =0 otherwise
*CP*	=*PERCATH* if *CATHOLIC* = 1; = 0 otherwise
*PROA8*_−1_	Proportion of total SOA births in the previous year to mothers from A8 countries
*RPCi*	Rotated principal components of 2001 Census socio-economic variables at SOA level; *i* = 1, …, 4
*PROL01*	Proportion of the SOA population with educational levels 0 and 1
*PROL45*	Proportion of the SOA population with educational levels 4 and 5
*DL01*	=1 if woman has educational level 0 or 1, =0 otherwise
*DL45*	=1 if woman has educational level 4 or 5, =0 otherwise

* Age of woman is divided by 30 to ensure the variables in the logit have similar orders of magnitude

The coefficients of the period and cohort effects in (1) are expressed as contrasts with the omitted base categories, 1997 for the period effects and the cohort born 1953–1957 for the cohort effects. (1) is considered an adequate predictor of the probability that a woman with the observed characteristics has a birth in a particular year (see Wooldridge 2009: 312–313 for a statement of such an approach).^1^ The object of the analysis is an estimate of the average marginal effect of religion, and individual coefficient values, apart from those involving the religion dummies, are not central.

The set of variables *DEMOG* covers the variables measuring age, parity and duration which collectively are considered the demographic profile of the particular community. If the fertility regime of Protestants and Catholics was the same, then the estimated coefficients for the variables included in *DEMOG* should not be statistically different which implies that the dummies establishing the religion of the woman should not be significant.

The probability that a woman gave birth in any year will be a function of her characteristics in the demographic profile but will not be completely determined by it. The time dummies encapsulated in *PERIOD* are free to vary each year. In effect this means that the constant term in (1) can change every year.

Given the informal nature of the model, robust standard errors were employed, based upon observations clustered at the individual level.

The test procedure consists of estimating a general model where all the coefficients relating to Catholics are allowed to differ from Protestants using dummy variables. Differences in the fertility behaviour of the two communities are then established by testing for the significance of such coefficients.

### Estimating the Effect of Religion

The key concept in attempting to quantify the effect of religion is the marginal effect (see, for example, Greene 2012: Chap. 17). Given the nonlinearity of the model, the marginal effect will depend upon the values of the independent variables of each individual. Since *CATHOLIC* is a dummy variable, the marginal effect is measured as the difference of two probabilities. If the variables other than *CATHOLIC* for the *i*th individual are summarized by the vector $$\overset{\lower0.5em\hbox{$\smash{\scriptscriptstyle\smile}$}}{X}_{i}$$, then the marginal effect of religion, *ME*_*CATHOLIC*_, for the *i*th individual is given by

2 $$ME_{CATHOLIC,i} = \hat{P}(BIRTH_{i} = 1\left| {\overset{\lower0.5em\hbox{$\smash{\scriptscriptstyle\smile}$}}{X}_{i} ,CATHOLIC = 1} \right.) - \hat{P}(BIRTH_{i} = 1\left| {\overset{\lower0.5em\hbox{$\smash{\scriptscriptstyle\smile}$}}{X}_{i} ,CATHOLIC = 0} \right.)$$

where $$\hat{P}$$ is the estimated probability of the woman giving birth conditional on her possessing the characteristics given by $$\overset{\lower0.5em\hbox{$\smash{\scriptscriptstyle\smile}$}}{X}_{i}$$. Note in (2) that *CATHOLIC* interacts with all (at least in the complete model) the explanatory variables and so the estimated coefficients for the variables $$\overset{\lower0.5em\hbox{$\smash{\scriptscriptstyle\smile}$}}{X}$$ in (2) will in general be different between the two terms on the right-hand side.

In (2), the marginal effect is calculated for an individual. The overall marginal effect is taken as the mean of the individual ones, which follows Jones et al. (2007), and allows the variation of the individual marginal effects to be displayed by age or time.

## Results

The findings of study are presented  in six parts. The first part constitutes a graphical analysis of the fertility panel. The second examines the variables employed in the analysis. The next four parts contain the results of the logit analysis. It starts with an examination of fertility differences between Protestants and Catholics. The next section uses these results to compute the marginal effect of being a Catholic. In the fifth part, the fertility of both religious groups is compared separately to those who share the same background but have no current affiliation. Finally, the differences between those with no current allegiance and those currently affiliated to their former one are examined.

### The Graphical Analysis

**Fig. 2:**
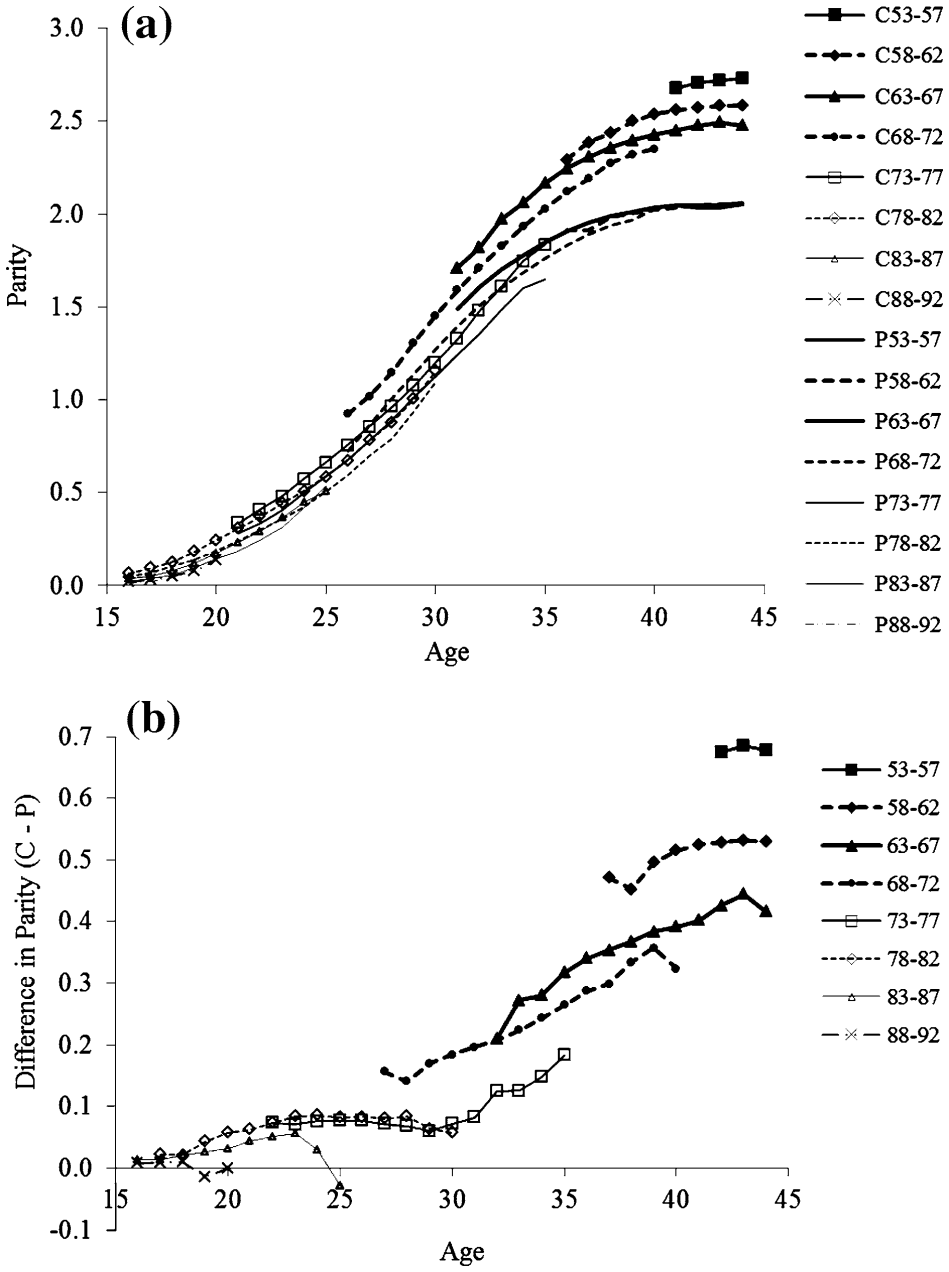
Average parity of women in the fertility panel by age, cohort and religion. **a** Cohorts, **b** differences by cohort. *Source*: NILS

In Fig. 2, average parity and age are graphed by cohort and religion. Cohort declines in fertility are marked for older Catholics, whereas similar Protestant cohorts were unchanged. In Fig. 2a, the average number of live births per woman is given by age, religion and cohort. In Fig. 2b, the differences in the two religious groups are graphed. When considering the cohorts born in 1953–1957, 1958–1962, 1963–1967 and 1968–1972, it can be noticed for Protestants, where the cohorts overlap in age, these are close together. The contrast with the equivalent Catholic cohorts is notable, with more recent cohorts having distinctly fewer children among this group. Furthermore, the fertility rate of Catholics is appreciably higher than Protestants. Looking at women of 40 years old, a continuous fertility decline is observed for Catholic women born 1958–1962, 1963–1967 through 1968–1972 going from 2.54, 2.43 to 2.35 children on average, whereas the corresponding figures for Protestants are: 2.02, 2.04 and 2.03 and thus stable over cohorts. A similar picture arises for 30-year-old women even though fertility levels are lower due to uncompleted fertility histories at that age. For younger cohorts, the pattern is similar but the differences are less marked.

### The Variables Employed in the Statistical Analysis

The variables employed in the statistical model were  defined in Table 1 (descriptive statistics), while they are broken down by religious status and presented in Table 2. Of particular interest in the latter is the substantial religious differential in the mean value of *RPC1* and *RPC2*. This reflects the recognized historical inequality between the two communities (see the CAIN website for an extensive bibliography).

**Table 2:** Descriptive statistics of the explanatory variables

Variable	Catholics		Protestants	
	*CATHOLIC* = 1	*f C* = 1	*PROTESTANT* = 1	*f P* = 1
*BIRTH*	0.064	0.060	0.057	0.057
*AGE*	0.999	1.020	1.030	1.028
*PAR0*	0.457	0.454	0.438	0.451
*PAR1*	0.140	0.166	0.152	0.180
*PAR2*	0.170	0.190	0.238	0.222
*DUR04*	0.604	0.536	0.532	0.506
*DUR* > *4*	0.506	0.532	0.545	0.546
*DUMDUR*	0.129	0.147	0.133	0.151
*PER1998*	0.088	0.093	0.092	0.095
*PER1999*	0.089	0.093	0.092	0.095
*PER2000*	0.090	0.094	0.093	0.095
*PER2001*	0.091	0.094	0.093	0.094
*PER2002*	0.092	0.094	0.092	0.093
*PER2003*	0.092	0.092	0.091	0.091
*PER2004*	0.092	0.089	0.090	0.088
*PER2005*	0.093	0.088	0.089	0.087
*PER2006*	0.092	0.086	0.088	0.085
*PER2007*	0.092	0.085	0.087	0.083
*C83t87*	0.137	0.118	0.112	0.102
*C78t82*	0.151	0.149	0.132	0.146
*C73t77*	0.160	0.163	0.158	0.180
*C68t72*	0.181	0.196	0.194	0.201
*C63t67*	0.182	0.189	0.200	0.192
*C58t62*	0.106	0.119	0.125	0.114
*C53t57*	0.027	0.026	0.031	0.030
*PP*	0.000	0.000	0.779	0.000
*PROA8*_−1_	0.023	0.031	0.027	0.030
*RPC1*	0.377	0.089	−0.159	−0.223
*RPC2*	0.147	0.534	−0.166	0.088
*PERCATH*	0.710	0.541	0.221	0.230
*N*	431,825	31,936	429,077	67,487
*PROL01*	0.239	0.221	0.246	0.229
*PROL45*	0.056	0.064	0.047	0.050
*DL01*	0.483	0.451	0.496	0.470
*DL45*	0.231	0.278	0.197	0.217
*N*	28,503	2270	30,609	4922

### Fertility Differences Between Protestants and Catholics

Equation (1) was estimated for the fertility panel using the variables defined in Table 1 for Protestants and Catholics, and the results are presented in Table 3. The hypothesis that all the Catholic and Protestant parameters are the same in this model is rejected by a Likelihood Ratio Test (LRT). A single logit was then run [based on (1)] that permitted all the coefficients to vary between the two groups through the repeated use of a *CATHOLIC* dummy variable. The results are presented on the left-hand side of Table 3. A more parsimonious model was generated by removing the insignificant coefficients (see Kennedy 1998), reducing the number of variables from 71 to 49. The dropped variables are from the *PERIOD* and *LOCALITY* sets and predominantly relate to the Catholic subset (period and cohort variables were only dropped if the entire set was insignificant). The discussion below centres first on the variables that were dropped and then interprets the remaining ones.

**Table 3:** Results from the logit estimation of *BIRTH* 1997–2007

*BIRTH*	General		Reduced	
	Column A	Column B	Column A	Column B
*DEMOG*				
*AGE*	60.879***	−1.081	52.198***	14.587***
		(5.098)	(7.475)	
	(11.495)			(1.748)
*AGE* ^*2*^	−69.069***	−10.971	−54.625***	−37.189***
	(18.186)	(23.972)	(11.965)	(4.251)
*AGE* ^*3*^	37.830***	15.525	27.690***	33.986***
				(3.945)
	(12.505)	(16.535)	(8.359)	
*AGE* ^*4*^	−9.363***	−5.594	−6.768***	−10.327***
	(3.158)	(4.186)	(2.151)	(1.244)
*PAR0*	0.623***	−0.225***	0.646***	−0.270***
	(0.036)	(0.047)	(0.032)	(0.038)
*PAR1*	1.051***	−0.186***	1.053***	−0.193***
		(0.034)		
	(0.026)		(0.026)	(0.034)
*PAR2*	0.087***	0.193***	0.088***	0.189***
	(0.027)	(0.035)	(0.027)	(0.035)
*DUR04*	0.965***	−0.149***	0.931***	−0.089***
	(0.029)	(0.038)	(0.025)	(0.030)
*DUR04* ^*2*^	−0.228***	0.037***	−0.221***	0.025***
		(0.010)		
	(0.007)		(0.007)	(0.009)
*DUR* > *4*	−0.140***	−0.101**	−0.196***	
		(0.046)		
	(0.034)		(0.023)	
*DUMDUR*	0.545***	−0.068	0.508***	
	(0.031)	(0.042)	(0.021)	
*PERIOD*				
*PER1998*	0.046	−0.011	0.040*	
	(0.031)	(0.043)	(0.021)	
*PER1999*	−0.011	−0.036	−0.029	
		(0.044)		
	(0.032)		(0.022)	
*PER2000*	−0.081**	−0.022	−0.093***	
	(0.034)	(0.048)	(0.024)	
*PER2001*	−0.139***	−0.021	−0.149***	
	(0.038)	(0.052)	(0.026)	
*PER2002*	−0.048	0.000	−0.048	
			(0.029)	
	(0.042)	(0.058)		
*PER2003*	0.018	−0.064	−0.015	
			(0.031)	
	(0.045)	(0.062)		
*PER2004*	0.088*	−0.108	0.031	
	(0.049)	(0.067)	(0.033)	
*PER2005*	0.087*	−0.069	0.051	
		(0.072)		
	(0.053)		(0.036)	
*PER2006*	0.147***	−0.126	0.081**	
	(0.057)	(0.078)	(0.039)	
*PER2007*	0.179***	−0.085	0.134***	
	(0.061)	(0.084)	(0.042)	
*COHORT*				
*C88t92*	0.290	−0.860**	0.460*	−1.197***
		(0.343)		
	(0.270)		(0.241)	(0.265)
*C83t87*	0.510**	−0.703**	0.670***	−1.018***
	(0.254)	(0.320)	(0.230)	(0.256)
*C78t82*	0.647***	−0.679**	0.782***	−0.944***
	(0.239)	(0.298)	(0.221)	(0.252)
*C73t77*	0.736***	−0.729**	0.841***	−0.934***
	(0.228)	(0.282)	(0.215)	(0.251)
*C68t72*	0.770***	−0.784***	0.849***	−0.940***
		(0.269)	(0.211)	
	(0.220)			(0.250)
*C63t67*	0.665***	−0.774***	0.722***	−0.889***
			(0.208)	(0.248)
	(0.214)	(0.259)		
*C58t62*	0.650***	−0.758***	0.681***	−0.825***
		(0.251)		
	(0.208)		(0.204)	(0.245)
*LOCALITY*				
*CP*	0.249**			
	(0.125)			
*CP* ^*2*^	−0.187*			
	(0.102)			
*PP*	−0.134		−0.091***	
	(0.185)		(0.033)	
*PP* ^*2*^	0.049			
	(0.140)			
*PROA8*_−1_	0.320**	−0.197	0.239**	
	(0.142)	(0.193)	(0.095)	
*RPC1*	0.047***	0.002	0.051***	
	(0.006)	(0.008)	(0.004)	
*RPC2*	−0.047***	−0.015**	−0.046***	−0.017***
	(0.006)	(0.007)	(0.005)	(0.006)
*RPC3*	0.010	−0.006		
	(0.008)	(0.011)		
*RPC4*	0.001	−0.005		
	(0.008)	(0.010)		
*CONS*	−23.805***	3.032	−22.056***	
	(2.649)	(3.463)	(1.706)	
*N*	860,902		860,902	
*R* ^*2*^	0.1036		0.1036	
*Log likelihood*	−175,695.9		−175,709.04	

Column A gives the estimated results for the variables in the first column

Column B gives the estimated results for the variables in the first column * *CATHOLIC*

The figures in parentheses are robust standard errors

*, **, *** indicate the coefficient is significant at the 10, 5, 1 % level

The insignificance of specifically the Catholic set of *PERIOD* variables is an important result as it demonstrates that the two communities responded in similar ways to shocks suffered by the Northern Ireland economy. It thus suggests that the forces that stimulated the revival of fertility early in the twenty-first century affected religious groups in a similar fashion. The proportion of A8 migrants in a locality did affect domestic fertility but in a similar fashion in both communities.

Secondly, the Northern Ireland economy changed appreciably in the period 1997–2007, but this development had a similar effect on the two communities given that the period effects did not differ. The correlation between the estimated period effects and the female activity rate is positive and strong (the correlation coefficient between the two series is 0.82).^2^ A positive relationship between fertility and female economic activity has been found in other OECD countries from the late 1980s (Ahn and Mira 2002; Rindfuss et al. 2003).

It is possible that a woman’s religiosity would be influenced by the proportion constituted by her co-religionists in her particular locality. The only variable that is significant is *PP*, the proportion of Protestants in the locality if the woman herself is a Protestant. Its effect on the latent index is negative which suggests that the more numerous Protestants are in a locality, the lower is their fertility. *CATHOLIC*, the religion dummy variable, on its own was insignificant and thus dropped. This implies that the constant in the parsimonious model is the same for Catholics and Protestants. Thus, there is no general increase in Catholic fertility, but rather it is restricted to those variables that are significant in the final column of Table 3.

In contrast to the above variables where the Catholic dummy was insignificant, the cohort variables were found to be highly significant and indicate that differential decline in Catholic cohort effects increased for women born between 1958 and 1967 and then plateaued before increasing again for those born 1983–1992. That the demographic profile differs between the two communities is clearly demonstrated by the highly significant differentials for Catholics across all the variables. The effect of this can be best viewed through considering the marginal effects.

### The Marginal Effect on Fertility of Being Catholic

The individual marginal effect is defined as the difference in the predicted probabilities of a woman with a particular set of characteristics having a child in a particular year when her religion is changed from Protestant to Catholic. The average marginal effect of being a Catholic, *ME*_*Catholic*_, is reported in Table 4, and across the entire sample is 0.0025, with a standard error of 0.0006. The mean is 4 % of the average proportion of women who have a birth in any particular year. The peak of *ME*_*Catholic*_ is 0.0051 which occurs when the woman is 29. Except for a slight decline at ages 20 and 21, the marginal effect steadily increases to 29 and then declines to near zero by age 44. Thus, the fertility differences between the two communities follow their demographic profiles with the Catholic one being slightly above the Protestant one.

**Table 4:** Means and standard errors of marginal effects

Marginal effects	Mean	Standard error^a^
cC,cP	0.0025	0.00060
fC,cC	−0.0033	0.0001
fP,cP	−0.0017	0.0001
fC,fP	0.0013	0.0004

^a^The calculation of standard errors follows Greene (2012) section 17.3.2

**Fig. 3:**
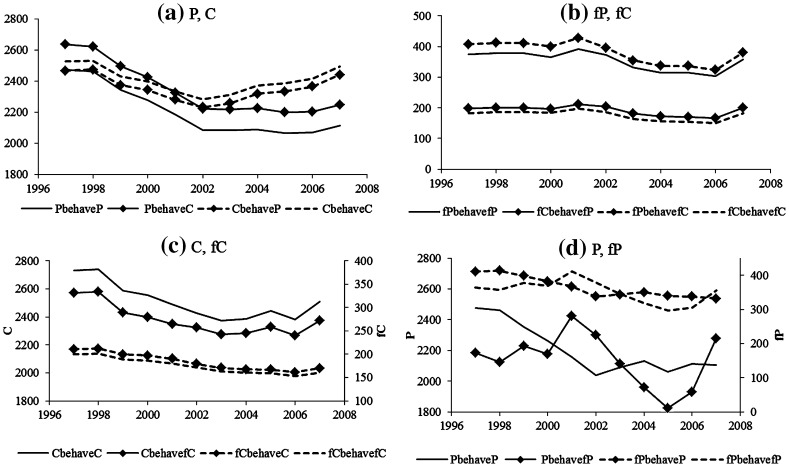
Predicted total births under different religious behaviours

**Fig. 4:**
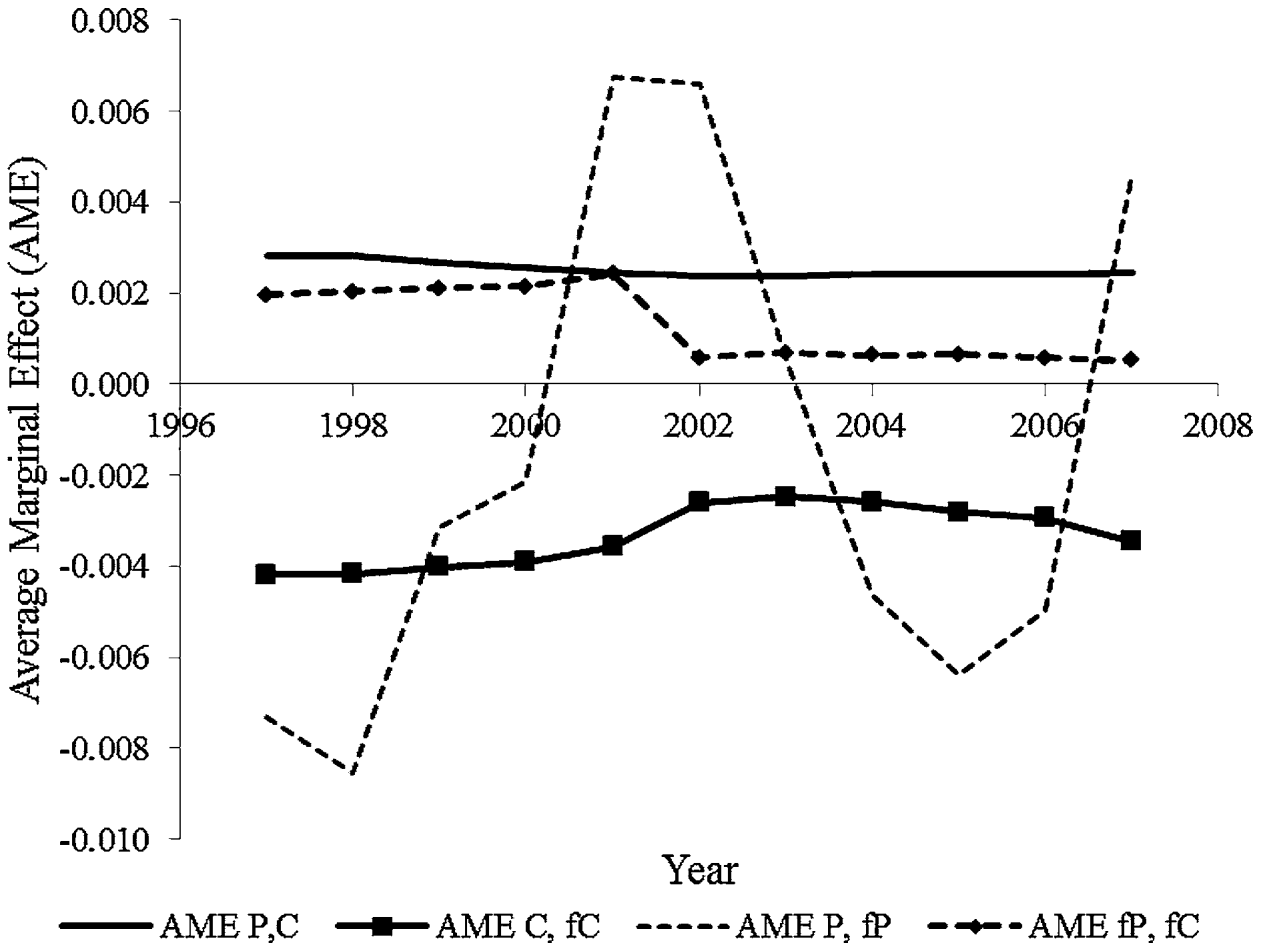
Average marginal effects by year

Further evidence on the extent of convergence in fertility between the two religious groups is provided in Figs. 3 and 4. The sum of the predicted births across a particular community will equal the prediction of the sum, that is, the total number of births expected. Using the estimated probability that the woman had a birth if she was of opposite religion means that the resulting predicted total births be compared. This is interpreted as the difference in total births if Catholics (Protestants) behaved as Protestants (Catholics). The result of Catholics behaving as Protestants is a 2.3 % decrease in the number of births, while Protestants behaving as Catholics results in a 6.5 % increase. These findings are presented in Fig. 3a.

Again, the graph suggests that there was very limited convergence in fertility rates between 1997 and 2007. In Fig. 4, *ME*_*Catholic*_ is averaged by year and graphed, showing little variation. Catholic fertility is slightly but distinctly higher than that of Protestants, and this differential is stable in the new millennium.

### The Marginal Effects of a Woman Changing From a Declared Religion to Having No Religion

The methodology employed in the previous two sections has also been applied to test differences in fertility between current and former Catholics and Protestants. First, those who declare themselves as Catholics are compared to former Catholics. The equality of coefficients between the two logits is firmly rejected by the Likelihood Ratio Test, and it is possible to reduce the full model from 69 to 36 variables (full results can be found  in online Appendix 2*).

The mean marginal effect for the panel is −0.0033, which is greater in absolute value than that between self-declared Protestants and Catholics. Figure 3b shows that when each of the two groups behaves as the other, the predicted births when the group behaves as Catholic are consistently higher over time than when they behave as former Catholics. Figure 4 demonstrates that the average marginal effect appears to converge at the beginning of the millennium but then diverges again. Basically, Catholics and former Catholics form distinct groups in terms of fertility, even though they share the same background.

The differences between Protestants and former Protestants are more marked compared to the Catholic case. The Likelihood Ratio Test rejects the equality of coefficients, although the reduction process leads to only 13 variables being dropped. The mean marginal effect is −0.0017, though the median is 0.0010. Figure 3c shows that former Protestants have lower fertility than Protestants, although this graph and that of the annual average marginal effect in Fig. 4 appear quite erratic. As with Catholics, Protestants and former Protestants thus exhibit different fertility behaviours.

The final part of the analyses compares the fertility of former Protestants and Catholics. Secularization is considered an important element of the second demographic transformation (van de Kaa 1987). The test of equality of coefficients between these two models generates a Likelihood Ratio Test which is (just) significant at the 5 % level. The mean marginal effect is 0.0013 with a median of 0.0004. The course of the average marginal effect between former Protestants and former Catholics over the panel period, displayed in Fig. 4, gives a strong impression of convergence, with the value 2002 to 2007 being close to zero.

### Differences in Characteristics Between Those with Religious Affiliations and Those Without

Table 5 reports the results of two relevant logits. Since education is likely to play a central role, the samples are reduced to include only those that are aged over 23 years, when education is generally completed. Since religious belief is derived from the Census in the panel, the sample is restricted to the year 2001. The dependent variables are binary and distinguish between those individuals that are Protestant (Catholic) from those that currently have no religion but formerly were Protestant (Catholic). Education levels are split into three groups that are combinations of the Census categories.^3^ At the locality level, a variable combines the proportion of women in the lower groups 0 and 1 (*L01*) and another which combines the higher groups 4 and 5 (*L45*). This is reproduced at the individual level by the dummy variables *DL01* and *DL45*.

**Table 5:** Explaining the loss of religion

Variable	*f P*	*f C*
*AGE*	−0.410***	0.220
	(0.127)	(0.186)
*RPC1*	−0.140***	−0.050***
	(0.011)	(0.013)
*RPC2*	0.140***	0.230***
	(0.012)	(0.016)
*RPC3*	0.020	0.000
	(0.020)	(0.023)
*RPC4*	0.140***	0.030
	(0.018)	(0.023)
*PERCATH*	0.310***	−2.250***
	(0.092)	(0.106)
*PROL01*	−0.640	−1.490**
	(0.408)	(0.597)
*PROL45*	−2.520***	−3.250***
	(0.607)	(0.849)
*DL01*	0.240	0.780***
	(0.200)	(0.294)
*DL45*	0.620***	0.830***
	(0.147)	(0.202)
*CONS*	−1.420***	−1.500***
	(0.148)	(0.219)
*N*	35,531	30,773
*R* ^*2*^	0.0121	0.0719

The figures in parentheses are robust standard errors

*, **, *** indicate the coefficient is significant at the 10, 5, 1 % level

There are three striking results of these analyses (Table 5). Firstly, given that the pseudo-R^2^ is almost six times greater, the logit explaining former Catholics is considerably better than that explaining former Protestants. According to the 2001 Census, 14 % of Protestants by background declared they had no current religious affiliation compared to 8 % of Catholics. The second result of note is that the greater the proportion of the population of the neighbourhood made up of people with the same community background, the less likely a woman loses her religious affiliation. The final point relates to the role of education. For both Protestants and Catholics, the signs of the locality effects are negative and thus the larger the proportion of poorly or highly qualified women in it makes it less likely that the woman concerned will lose her religion. On the other hand, if a particular woman herself is lower or higher educated, then she is more likely to lose her religion. The marginal effect sheds light on this apparent contradiction.

The definition of the marginal effect of education on the probability of losing a religious affiliation has to take into account that there are three educational categories. The average change of a Protestant woman as she moves from the lowest educational attainment to the middle one (groups 0 and 1 to groups 2 and 3) is −0.0274 while moving to the highest groups, 4 and 5, from the middle has an average change of 0.0801. Thus, those with middling education are likely to be the most religious, while those at the extremes, with little or higher education, are more likely to lose their religious affiliation. For Catholics also, those with middling education are least likely to lose their religious affiliation with the marginal effects being −0.0466 moving up to middling education and 0.0604 moving beyond it. Over time, the increase in higher education is likely to have discouraged religious affiliation.

## Conclusion

The central issue tackled in this paper is the contribution of religion to fertility in contemporary Northern Ireland. To address this, the Northern Ireland Longitudinal Study has been employed which in this case matches the records of women from a sample of the Census and the Birth Registration Data. This results in the construction of a panel consisting of over one hundred thousand women in the period 1997–2007. Using these data has two major benefits. Firstly, the difference between groups in the population can be expressed quantitatively by the marginal effect estimated from regression analysis where the latter controls for differences in demographic and socio-economic structure between groups. The marginal effect thus expresses the differential fertility of subgroups of the population as a statistic. This can be disaggregated over time so that the extent of convergence between groups in the period considered can be assessed. Secondly, the NILS allows the definition of groups to be developed. This allows the effect of religion on fertility to be compared not just between Protestants and Catholics but also, to highlight contribution of religion by itself, by comparing Protestants with former Protestants and Catholics with former Catholics. Finally, comparing the fertility of former Protestants and Catholics provides insight into the extent of secularization in Northern Ireland.

The results show that the marginal effect of a being a Catholic woman is a 4 % higher fertility compared to a Protestant. This difference is small but significant though it is likely to fall in the future because there has been a marked decline in the fertility across older Catholic cohorts and these will drop out with the passage of time. Among younger cohorts, the difference between Catholic and Protestant fertility is less marked. Against this, there is no evidence of convergence for the panel period. No significant difference between the period effects of Catholics and Protestants is found. This suggests that economic and social change during the time examined impacted both communities in a similar way. Changes in the trade-off between childbearing and labour market participation have been put forward as an explanation of the recent fertility revival (Kalwij 2010). The picture then is one of both groups responding to external developments in a similar way but still maintaining distinctive demographic profiles.

Marginal effects are particularly informative when comparing those with religion to those without it. The magnitude of the marginal effect comparing Catholics with former Catholics is larger than that between Catholics and Protestants. This suggests that the pure fertility impact of religion in this case is considerable since both groups would have a similar background. When such an analysis is carried out on Protestants, the results are similar but less robust. Of particular interest then is the fertility behaviour of former Protestants and former Catholics. The marginal effect in this case is the smallest of the four comparisons though it is still just significant at the 5 % level. Over time the marginal effect declines and gives a firm indication of convergence.

The overall picture of fertility in contemporary Northern Ireland by these statistical results can be presented in terms of the groups concerned. Protestants and Catholics form the religious groups, and these are distinctive in a small but definite fashion. It is arguable when considering former Protestants and Catholics that they together comprise one group. While the marginal effect is just significant between the two sets, its magnitude is the smallest of the four sets considered. Moreover, over the period considered, the two birth rates show definite convergence. If a single group is designated, then it can be considered as secular. Secularism though is not a central element within the future of fertility in Northern Ireland. The group is small as it comprises only 13 % of the population in 2001. Moreover, the Northern Ireland Life and Times Survey gives no impression of a fall in religious commitment in the period considered. Given the higher birth rate of the religious groups, if anything secularism is under demographic pressure. In this it reflects a general European trend (Kaufmann et al. 2012).

Doctrine is the most obvious explanation of higher Catholic fertility given the natalist position of the Catholic Church compared to the more choice variety advanced by Protestants. Both groups though have experienced sharp falls in fertility for decades and indeed across the continent. Despite this, Catholic doctrine has hardly changed. According to McQuillan (2004), one element necessary to establish a credible role for religion in determining fertility is that the religious group must be capable of communicating its teachings and to enforce compliance. By the control of primary and secondary education (see Byrne and Donnelly 2006), the Catholic Church clearly can communicate its teachings, but the fertility evidence over decades is equally clear that it cannot enforce compliance.

According to Bongaarts and Watkins (1996: 665), social interaction is a critical process that should have a central role in any comprehensive and realistic theory of fertility. Within Northern Ireland voluntary residential and (at the primary and secondary level) educational segregation severely curtails social interaction. This means that though both the Protestant and Catholic groups have responded in a similar fashion to economic and social developments, the historical lack of interaction means that some distinction is maintained at least in fertility. It is significant that social interaction is greatest in the secular group. Those that lose their religion tend to live in localities where religious homogeneity is less than the religious groups and the socio-economic status is higher.

It is important to note that possession of religious belief is not linked linearly to education. The population layer most likely to have a religious faith comprises those with intermediate educational attainments. Those with lower and higher attainments are more likely to have lost them. Higher education is the most likely avenue to social interaction. The religious homogeneity of a locality though reinforces religious belief irrespective of a woman’s educational attainment.

There are a number of limitations to the analysis presented in this paper. Firstly, the NILS data are limited to that provided by the Census and thus do not give annual income. This has led to period and locality variables being used as substitutes. Period effects are significant but do not differ on the basis of religion and thus allow a single constant in the regression to change with economic circumstances. The effects are closely correlated with female activity rates. Also, data from the Census were used to construct the socio-economic characteristics of the locality in which the woman resided. The use of district averages as an indicator of individual variables is not totally satisfactory though the estimates of the particular locality parameters in the general statistical model used are not taken as measures of gradients at the individual level (Wooldridge 2002 reveals how rigorous the assumptions for this are). The locality variables are used pragmatically to improve the ability of the model to predict an individual birth. Basically, the use of NILS, like the use of any other data, involves a trade-off between quantity of available data and survey coverage.

Secondly, the role of education in the loss of religion is limited by the data relating to only 2001 and those aged above twenty-three by which time most women would have completed their education. An additional issue concerning the data is thus raised to those discussed in the previous paragraph. The interpretation of the role of social interaction in this model is complicated by the number of locality variables considered.

Thirdly, the nature of the religion variables used has limitations. This paper has considered the religion of current and former Catholics and Protestants. The latter group is an aggregation of over two hundred churches that have household members in the Census. Such aggregation clearly involves a loss of information, but it is necessary for tractability. Comparative fertility among the Protestant sects certainly though has potential for further research. The Census employs the self-declared religion of the household and does not investigate religiosity. Thus, the ‘a la carte Catholic’ is counted along with the member of Opus Dei as being Catholic. By way of contrast, the European Values Survey has seven categories concerning the attendance of the respondent at religious services. A similar though less pronounced problem exists with the concept of secularization. Here too the Census classifies those that do not have a religion by a simple binary classification. Against this, the study of secularism contained in Taylor (2007) is much more nuanced where the historical and philosophical environment that pertains plays a central role. Rather than change over time, the issue here is of a society where division is frequently expressed in terms of religion. The loss of religion has a social context, and it is not straightforward to discern the direction of causality.

It is, however, unlikely that the shortcomings listed above change the broad thrust of this paper. The challenges of determining the influence of religion on fertility were summarized over 40 years ago Goldscheider (1971) and are still relevant: ‘religion interacts with other aspects of social organization of the subcommunity and subculture, and it is through the total content of the social organization, which the particular theology is but one part and often not the most significant, that fertility patterns are affected’ (1971: 274).

## Electronic supplementary material

Below is the link to the electronic supplementary material.

Supplementary material 1 (PDF 497 kb)

## Footnotes

1 Education is generally completed by the time a person is 24 and thus excluding women aged below this and only considering 2001 allows educational attainment at an individual level (taken as whether the woman had a degree) to be regressed upon the *RPC* variables and *PERCATH* at the SOA level. Those correctly classified amounted to 79 % for the Catholic sample and 81 % for the Protestant. This gives some reassurance in the use of locality variables in the analysis.

2 The female activity rate is obtained from DETINI (2007).

3 These are defined in the Registrar General’s 2006 report, p. 38.
