# Supplementary material for: Religion and Fertility in Contemporary Northern Ireland
Source: Eur J Popul. 2016 Sep 28;32(4):599–622. doi: 10.1007/s10680-016-9399-8 (PMC5056953; doi:10.1007/s10680-016-9399-8)
Supplement: Supplementary file 1 — Supplementary material 1 (PDF 497 kb) [file 10680_2016_9399_MOESM1_ESM.pdf]

Data Appendix

The NILS

The sample frame of the NILS consists of all registrations held within the Northern Ireland Health Card registration system. From 2009 this has been administered by the Business Services Organisation (BSO), an agency of Health and Social Care (HSC) and formerly by the Central Services Agency. The database is continuously updated to take account of births, deaths and migration. The NILS consists of a 28.5% systematic sample taken biannually from this database on the basis of 104 confidential birthdays. This provides the date of birth, gender and location of each individual in the NILS sample. Much more extensive information is provided by linking the NILS sample to the census and, in particular, the 2001 one.

The NILS sample drawn from health card registrations was approximately 508,000 at the time of the 2001 census. Of these individuals approximately 449,000 were linked to the 2001 census records through a three stage process. The first of these extracted the records of those who had an exact electronic match of forename, surname, gender, date of birth and location. Fuzzy matching techniques with clerical assessment and confirmation were applied to the remainder. Finally, the remaining unlinked records were subject to a wholly manual linkage process (O'Reilly et al 2012).

The failure to link records has three causes. Firstly, the 2001 census was a 'one-number' one.[1] Under-enumeration of the population was estimated on the basis of a post census survey which led to an additional 4.8% of the total number actually enumerated being statistically imputed. It would be anticipated from this that there would be 22,500 such cases in the NILS sample which thus could not have a health card registration. Secondly, the health

[1] The 2001 Census used an approach (The One Number Census) to adjust the census database for the estimated under-enumeration so all statistics add up to 'One Number'. Thus if a household was not enumerated its size and characteristics were imputed (ONS, 2001).

card registrations are subject to 'list inflation' due principally to individuals who have emigrated without the health service being informed. It is estimated that this accounts for 4.7% of health card registrations or approximately 24,000 of the NILS sample. Such records cannot have associated census return. Finally there is a failure to match bone fide records which is estimated to amount to 12,000 cases (O'Reilly et al, 2012), a rate of 2.5% (12*100/(508-24)).

In Northern Ireland the registering of a birth is a legal obligation but, unlike the case of completing a census form, the incentives, such as access to health care and benefits, are overwhelmingly positive. The linkage methodology employed is similar to that of the census except there is an addition check to ensure that the mother's birthday corresponds to that on the health card. The results are examined in detail below.

The fertility panel

The fertility sample was derived from biannual downloads from the BSO for the years 2000 to 2007 of those women who had health card registrations and were aged 16 to 44 at the time of the download. The (unbalanced) fertility panel consists of those women present in any download who also have a census record. If a NILS woman had a birth in any year details of this event were supplied by the General Register Office (GRO). The GRO data actually extends from 1997 so an additional four annual observations were obtained by assuming a woman present in 2001 was also present 1997-2000. A birth event in any year was described by a binary variable which became the dependent variable in the regression analysis. Thus the fertility data brings together three data sets: the health card registration set which establishes the presence of the woman in Northern Ireland in any year; the GRO births data which indicates whether she had a birth in that year; the census data which provides details of the woman's characteristics in 2001 (though some information from the 1991 census was also employed).

The fertility panel may more accurately defined as the 2001 cohort of women aged between 10 and 48 whose census returns were successfully linked to the health card registration database. During the period of the study there was substantial immigration into Northern Ireland from the 8 countries that acceded to the EU in 2004. None of these immigrants would have completed a census return in 2001 and thus could not appear in the fertility panel. Consequently the panel describes the fertility behaviour of an overwhelmingly native cohort and appropriately it is within this cohort the influence of religion is being examined.

The construction of the panel was in steps, the first of which dealt with the births data. Each step was accompanied where possible by checks on consistency and representativeness which are described below.

The births data

These were obtained from the GRO and consisted of the births to mothers whose date of birth was one of the 104 NILS dates; information on the birth registration document was also included. For the period 1997-2007 this amounted to 70,571 births to mothers who were resident in Northern Ireland and who were linked to the health card database for that year. The data were checked for implausible fertility rates, taken as a woman aged 20 who had more than 4 babies, up to one aged 24 with more than 8; no women came into these categories.

For women who had two or more births over the period it was possible to run additional checks, namely whether the mother's age falls with increase in her parity and if the time between two births was greater than zero but less than six months. Together these led to 40 mothers (111 births) being dropped, 15 for whom there was less than 6 months between sequential births, which would have been treated as a miscarriage. There were 973 twins in the births data. This gives a live (only live births are considered in the analysis) twinning rate

of 1.40%. The tables that accompany the annual Registrar General's reports are available on the web for 2004-7 [2]and allow a slightly different live twinning rate (where the denominator is the number of confinements rather than the total number of births) to be calculated. In the 4 years considered this varied annually between 1.31% and 1.44%, so the twinning rate in the fertility sample is as would be anticipated.

In the initial examination of the births data the total number of previous births to the mother, which is a variable that appears on the GRO data, was checked for discrepancies, namely, whether total previous births did not equal total previous live births plus still births. This produced a large number of cases. When this was raised with the Demography and Methodology Branch of NISRA they responded by saying that the only check on total previous births was when the number was unrealistically high. They advanced several reasons for inconsistencies:

- Typing errors, as the number does not appear on the birth certificate and so is not checked by the person registering the birth.
- Registrars record the total previous in different ways, especially in the case of multiple births
- There is a reticence to raise the issue of stillbirths
- The person registering the birth is not the mother and may be misinformed of previous births
- If a birth occurs outside marriage and either of the partners has children from a previous relationship then they may only specify the children from the current relationship.

[2] Table 3.3 Multiple births (live and still), by age of mother, sex and type of confinement) for 2004-7. Obtained from [http://www.nisra.gov.uk/demography/default.asp132.htm].

Given this, the behaviour of total previous births was not employed as an indicator of data inconsistencies.

The project began with births data for 1997-2005 and this was augmented during the exercise as more data, 2006-07, became available. For the initial data it was possible to compare the NILS sample with those births that occurred in the same period to mothers who did not possess a NILS birthday. To ensure that the full births data was not disclosive, the data provided did not include an identifier for each mother; in addition the order of births was randomised; consequently neither multiple births nor births to the same mother could be identified. There were 145,273 births to non-NILS mothers who were resident in Northern Ireland, compared to 56,699 in the NILS; the NILS proportion was 28.1% as against the 28.5% expected.

The two sets of births were compared with respect to country of birth, the marital status of the mother and the mean age of the mother by parity as given by the total previous number of births. There were no systematic differences between the two sets in any of these cases apart from the proportions of the very small number of A8 cases in the country of birth table.

The independent variables in the regression

The independent variables employed in the regression analysis can be divided into distinct sets, namely those that depend on a linked census record and those that do not. The latter consist of the period and the cohort effects that are binary dummy variables and *AGE* which is taken from the BSO download and will exist for all cases in the sample. The variables that require a census link deal with parity (from which duration is calculated), location and religion. Although the completion of a census return is a legal obligation, forms are returned incomplete which entails NISRA imputing values for omitted responses. In the analysis imputed values were treated as missing. Given that the analysis was restricted to

subsamples, such as those who declared themselves as either Protestant or Catholic, the representativeness of the sample will depend on the distribution of the characteristics of the entire derived sample. The derived variables are examined in turn below:

Parity: For each person recorded on the census form the respondent completes a set of relationship questions between each of the members of the household. Thus for each woman aged 10 to 48 in the NILS sample at the time of the census, the census will contain the number of persons whose relationship to her is that of ‘mother’. The next box in the question includes ‘step-mother’. ‘Foster-mother’ is not a specific option and might fall into the categories ‘Other related’ or ‘Unrelated’ or, indeed, ‘mother’. The relationship of adopted children would probably be recorded as ‘mother’ and so would introduce a small number of errors into the parity estimates. The first approximation to a woman’s parity at the time of the census was given by the number of persons in the household to whom she was related to as ‘mother’.

It is possible that a woman in her forties in 2001 would have a child in the late teens who might have left home either permanently or as a student. It is possible to reduce any underestimation of her parity because of this if the woman was also linked to the 1991 census records. Question 7 in the 1991 census asked the number of children born alive to any woman in the household who was married, widowed, separated or divorced. This potentially provides an alternative parity estimate.

The information on the birth registration form allows the generation of further estimates of parity. If the woman has more than one birth recorded then the parity from the first after census day 2001 is taken to be her parity then; if all her recorded births are before census day then the parity on that day is taken as the one closest to it. Another estimate is generated by simply adding the number of births recorded between 1997 and census day. The final estimate consists of adding the latter number to the 1991 census estimate. In the first

instance the estimated parity from the 2001 census was taken; should any of the alternative methods produce a higher estimate, then the highest of these replaced that of the 2001 census.

The source of the parity estimate for the fertility sample is shown in Table DA.1. In over 90% of the cases, whether by records or women, it was the 2001 Census. In terms of the number of women in the sample, the 1991 Census was the next most important source (5.2%), though by the number of records it was the birth registrations (3.6%).

Location: The smallest geographical unit identified in the NILS is the Super Output Area (SOA) of the Census of which there are 890, giving an average population of about 1900. In terms of records (women) there are 1,014,961 (127,607) in the NILS sample.

Religion: The respondent to the census questionnaire first answers the question ‘Do you regard yourself as belonging to any particular religion?’ If the answer is ‘yes’ then the respondent is directed to a question concerning denomination – the categories are ‘Roman Catholic’, the three main Protestant religions and ‘Other’. If the latter response is given then the respondent is asked to write in the particular religion concerned. If the respondent indicates that she does not belong to any religion then she is directed to a question concerning the religion she was brought up in – the possible responses mirror those of the previous question but now also include ‘None’.

There is an issue concerning the definition of ‘Protestant’. In the published Census tables the numbers of the principal Protestant religions, the Presbyterian Church in Ireland, the Church of Ireland and the Methodist Church in Ireland are presented individually; there is an additional category ‘Other Christian (including Christian related)’ where sizeable numbers of Baptists, Brethren, the Congregational, the Elim, Free Presbyterian and the Pentacostal Churches are aggregated as well as those who record their religion as ‘Christian’. To facilitate comparison with the census, those whose religion fell in this category or within the principal Protestant religions were classed as ‘Protestant’. Those women who considered

themselves either Roman Catholic or belonging to one of the principal Protestant churches constituted 93% of the fertility panel in 2001.

In Table DA.2 a subset of the responses are presented to illustrate how sample size is affected by the responses to the religion questions. The number of missing records (173,353) is the same as that for the locality question but in addition there are 20,956 non-resident students and 5,205 cases where the religion has been imputed. Respondents in these three categories do not appear in the fertility panel. Of those women who did not belong to any religion about a quarter were brought up as Roman Catholics.

The fertility panel: There are 1,188,314 records relating to 157,257 women in the NILS sample. To appear in the logistic regression on births, each woman must have records on parity, location and religion. In Table DA.3 the size of each of these three sets is presented and shows that religion is the smallest. The intersection of these three sets defines the fertility panel used in the analysis and it consists of 983,017 records and 123,455 women though only just over one thousand women are in the religion set but not in the intersection of all three sets.

The representativeness of the fertility panel: This will be revealed by comparing the characteristics of the fertility panel to that of the population, effectively as revealed by the 2001 census. The fertility panel is larger than the sum of those women classified as *CATHOLIC, PROTESTANT, fC* and *fP* but the proportions of each of these groups in the fertility panel in 2001 should be similar to those in the population as recorded by the census. For that year there were 90,810 women in the fertility panel. It would be desirable to compare both parity and religion, broken down by age, to the population data. Unfortunately parity is not covered by the 2001 census and consequently the parity estimates produced in this paper are based on a sample considerably larger than that in any sample survey. Thus in relation to

parity no gold standard exists and so attention is restricted to religion. Given that difference in fertility across religions is the focus of this paper, the limitation is not unduly restrictive.

In Table DA.4 the distribution of religion across the fertility panel in 2001 is compared to that of the 2001 Census. Rows A and B give the distribution of Catholics and Protestants in respectively the Census and the fertility panel with the proportions in row C. The fertility panel is 25.6% of the census total as opposed to the 28.5% NILS sampling fraction. Given that the enumerated census total is expanded by 4.8%, the sample fraction is 26.9% of the enumerated total. The remainder of the difference can be put down to list inflation and linkage failure. The expected number of the two religions is then their census totals multiplied by overall actual sampling fraction, 0.25647, and this appears in row D. The difference between the actual and expected number of women in each religion is given in row E and this is expressed as a percentage in row F. Thus in the fertility panel in 2001 there are 0.5% fewer Catholics than expected and 3.6% more Protestants. Adjusting these figures for the differential rates of imputation, 0.09% for Catholics and 0.16% for Protestants, leads to no substantial change: -0.55% for Catholics and 3.52% for Protestants. Thus there are slightly more Protestants than expected in the fertility sample and very close to the anticipated number of Catholics.

In Fig. DA.1 the sampling fractions for the two religions are broken down by age and displayed. While, as would be expected given Table DA.4, the Protestant graph lies above that for Catholics for the aggregated ages, the trends over age mirror each other fairly closely. Thus there is no evidence that the sample proportions trend differently over age for the two religions.

How is the slight over-representation of Protestants in the sample likely to affect the results in this paper? First consider the parameter estimates: the general model results in Table 3 illustrate that all the parameter estimates are allowed to vary freely between Catholics

and Protestants so the slight over-representation of Protestants is unlikely to impose any major bias to the results. This is particularly relevant as the focus of this paper is upon the marginal effect on fertility of religion where the parameter estimates for each religious group will exert little influence on the other. Secondly, the population totals for the various religions will include a 4.8% imputation which introduces errors into the population totals as recorded in the census. The size and direction of these errors is unknown. Given the above, it is reasonable to employ the fertility panel in the statistical analysis.

Table DA.1
Sources of the Parity Variable in the Fertility Panel

Column	Source	Records		Women	
		Number	%	Number	%
A	2001 Census	914,213	93.0	113,240	91.7
B	1991 Census	30,593	3.1	6,406	5.2
C	Birth registrations	35,664	3.6	3,558	2.9
D	Number of births, 1997-2001	976	0.1	93	0.1
E	B+D	1,571	0.2	158	0.1
	Total	983,017	100.0	123,455	100.0

Table DA.2
Selected Responses to the Religion Question in the NILS Sample

Response	Records	Women
Catholic	434,578	54,731
Non-resident student	20,956	2,528
Imputed	5,205	652
None	120,389	15,063
(of which former Catholics)	34,098	3,966
Missing	173,353	29,650
Total	1,188,314	157,257

Table DA.3
The Sets of Variables used in the Fertility Panel

Variables relating to:	Non-missing records	Non-missing women
Parity	1,060,962	133,244
Religion	988,800	124,427
Location	1,014,961	127,607
Intersection	983,017	123,455

Table DA.4
The Actual and Expected Religious Distribution in the Fertility Sample

Row		Total	cC	cP
A	Women 16-44 Census	354076	154641	149994
B	Women 16-44 Fertlity Panel 2001	90,810	39450	39864
C	Panel proportion of census subtotals	0.25647	0.25511	0.26577
D	Expected women given Total proportion = A*0.25647		39661	38469
E	Actual - Expected		-211	1395
F	% error = E*100/D		-0.53	3.63

Figure DA.1
Protestants and Catholics in the Fertility Sample as a Percentage of the Census Totals by Age

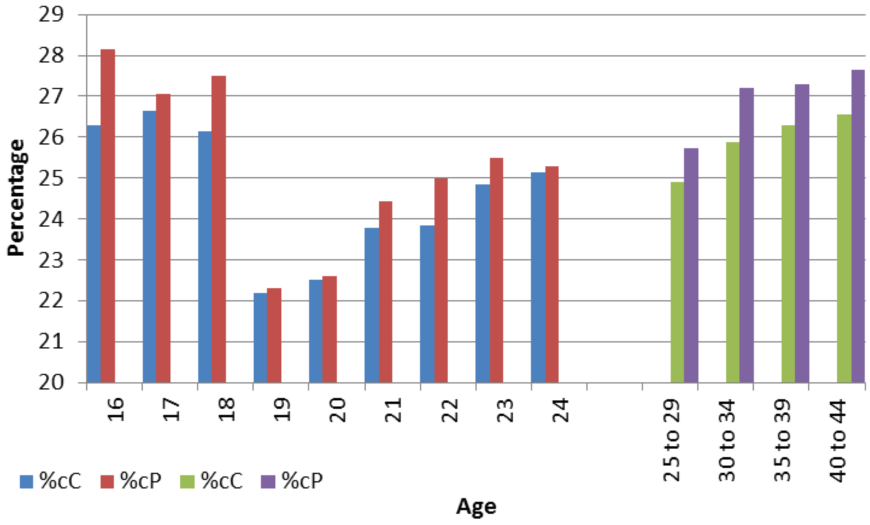

Appendix 2* Results from Logits Incorporating Former Religious Adherents

BIRTH	C & fC		P & fP		fP & fC	
DEMOG						
AGE	63.503***	11.845***	60.876***	18.154***	89.863***	
	(9.432)	(2.154)	(10.362)	(3.404)	(19.985)	
AGE^2	-86.230***	-32.884***	-69.511***	-46.312***	-136.454***	
	(15.064)	(6.842)	(16.390)	(8.224)	(32.148)	
AGE^3	57.776***	29.307***	38.330***	40.070***	95.181***	
	(10.447)	(7.051)	(11.283)	(7.502)	(22.466)	
AGE^4	-16.101***	-8.512***	-9.542***	-11.378***	-25.721***	
	(2.658)	(2.363)	(2.853)	(2.353)	(5.754)	
PAR0	0.380***		0.606***	-0.318***	0.203***	
	(0.029)		(0.034)	(0.059)	(0.066)	
PAR1	0.850***		1.041***	-0.241***	0.773***	-0.117*
	(0.021)		(0.025)	(0.050)	(0.054)	(0.065)
PAR2	0.268***		0.073***		0.039	
	(0.021)		(0.025)		(0.051)	
DUR04	0.807***		0.958***	-0.190***	0.708***	
	(0.024)		(0.028)	(0.060)	(0.053)	
$DUR04^2$	-0.189***		-0.226***	0.040**	-0.172***	
	(0.006)		(0.007)	(0.017)	(0.014)	
DUR>4	-0.245***		-0.142***		-0.152**	-0.173***
	(0.030)		(0.031)		(0.069)	(0.060)
DUMDUR	0.471***		60.876***		0.397***	
	(0.028)		(10.362)		(0.062)	
PERIOD						
PER1998	0.039		0.049	-0.027	0.034	
	(0.028)		(0.031)	(0.084)	(0.063)	
PER1999	-0.046		-0.005	0.087	0.010	
	(0.029)		(0.031)	(0.084)	(0.064)	
PER2000	-0.104***		-0.072**	0.120	-0.052	
	(0.032)		(0.033)	(0.091)	(0.066)	
PER2001	-0.159***		-0.126***	0.295***	0.006	
	(0.035)		(0.035)	(0.096)	(0.069)	
PER2002	-0.043		-0.039	0.300***	0.088	
	(0.038)		(0.038)	(0.105)	(0.077)	
PER2003	-0.050		0.030	0.168	-0.009	
	(0.041)		(0.040)	(0.114)	(0.082)	
PER2004	-0.022		0.103**	0.055	-0.031	
	(0.045)		(0.042)	(0.123)	(0.087)	
PER2005	0.025		0.106**	0.010	-0.012	
	(0.048)		(0.045)	(0.133)	(0.092)	
PER2006	0.020		0.170***	0.043	-0.004	
	(0.052)		(0.047)	(0.143)	(0.097)	
PER2007	0.105*		0.204***	0.230	0.218**	
	(0.055)		(0.050)	(0.150)	(0.099)	

BIRTH	C & fC		P & fP		fP & fC	
COHORT						
C88t92	-0.562***			-0.751		
	(0.205)			(0.553)		
C83t87	-0.190		0.244***	-0.624	0.261**	
	(0.188)		(0.058)	(0.506)	(0.111)	
C78t82	-0.017		0.398***	-0.448	0.524***	
	(0.172)		(0.063)	(0.463)	(0.122)	
C73t77	0.020		0.506***	-0.632	0.423***	
	(0.160)		(0.073)	(0.431)	(0.143)	
C68t72	-0.010		0.557***	-0.744*	0.281*	
	(0.150)		(0.085)	(0.406)	(0.167)	
C63t67	-0.108		0.469***	-0.619	0.213	
	(0.142)		(0.098)	(0.384)	(0.195)	
C58t62	-0.103		0.475***	-0.839**	0.052	
	(0.135)		(0.116)	(0.371)	(0.231)	
LOCALITY						
CP					-0.017	
					(0.084)	
CP^2						
PP					-0.235***	
					(0.062)	
PP^2						
PROA8	0.133		0.398***		0.465*	
	(0.123)		(0.130)		(0.246)	
RPC1	0.053***		0.052***		0.044***	0.040**
	(0.004)		(0.005)		(0.013)	(0.020)
RPC2	-0.064***	0.041***	-0.049***		-0.048***	0.017
	(0.005)	(0.014)	(0.005)		(0.012)	(0.020)
RPC3					0.007	-0.043
					(0.016)	(0.029)
RPC4					0.026	-0.033
					(0.018)	(0.032)
CONS	-21.486***		-23.529***		-25.828***	
	(2.147)		(2.391)		(4.522)	
N	463761		496564		99423	
R^2	0.0943		0.1070		0.0789	
Log likelihood	-99577.007		-96510.12		-20376.968	
LRT (critical value)	128.81 (53.38)		251.92 (53.38)		55.49 (53.38)	
Wald (critical value)	38.32 (47.40)		20.83 (22.36)		41.28 (42.56)	

The LRT relates to the test of equality of all coefficients in the full model; the critical value is that for a χ^2 variable with 38 df and a significance level of 0.05.

The Wald relates to the reduction of variables test; the critical value is that for a χ^2 variable with degrees of freedom equalling the number of variables dropped and a significance level of 0.05 (see Table 3 for clarification).

Appendix 1*

The construction of measures of locality

The lack of economic variables at an individual level was ameliorated by the inclusion of variables that described the locality that the individual was resident in at the time of the 2001 census. The locality was taken as the Census Super Output Area (SOA) of which there were 890 in 2001 with an average number of persons in each of 1900. The variables selected on the basis of their demographic and socio-economic relevance are described in Table A1*.1.

The proportion of persons from a Catholic community background is employed as a separate variable in the analysis because of its role in fertility and so is not included in the principal component reduction. The socio-economic status of the locality is proxied by the educational, social grade and rented variables; the demographic structure by the median age of the population and the proportion of households, married, cohabiting and lone parent, who have dependent children together with the proportion of women 16-74 who are economically inactive but are looking after home or family.

There are thus eleven variables that summarise the relevant characteristics of the locality. These are reduced by principal components and the first four components together account for over 90% of the variance in the data, as indicated in Table A1*.2. These four components were rotated using a Varimax rotation to facilitate interpretation. The resulting loadings are given in Table A1*.3.

The component *RPC1* is clearly inversely related to potential economic wellbeing – the proportion of individuals without qualification, the female inactivity rate, the proportion of lower social classes and lone parents with dependent children all load positively on it,

while the proportion with degrees loads negatively. Among the four rotated components *RPC1* is the only one where education registers substantially. As shown in Table A1*.2, *RPC1* accounts for a third of the variance in the locality variables.

RPC2 is inversely related to social status as the proportion of social grades D and E load positively on it, as does rented and the proportion of single parent households; the median age is positively related while the proportion of married households with dependent children is negatively, suggesting an older, poorer population.

RPC3 is positively related youth with the highest (absolute) weighting to median age and the proportion of single households; *RPC4* is positively related to non-traditional lifestyles, highlighting cohabitation and lone parenthood.

The proportion of persons from a Catholic community background, PERCATH, is employed as a separate variable in the analysis because of its central role. The results of regressing PERCATH on the rotated components are presented below (where the figures in parentheses are t ratios):

$$\underset{(51.15)}{PERCATH = 0.430} + \underset{(3.43)}{0.019RPC1} - \underset{(4.78)}{0.028RPC2} + \underset{(24.80)}{0.165RPC3} - \underset{(8.83)}{0.076RPC4}$$

$$N = 890 \qquad F = 189.1 \qquad R^2 = 0.46$$

Less than half of the variation in the religious composition of the SOA is explained by the rotated components which suggests that the religious representation in a locality is not simply a function of its socio-economic structure.

Table A1*.1 2001 Census variables employed at the SOA level

Variable	Definition	Table
popnoqual	% population with no or low levels of qualifications	KS13
popdeg	% pop with education equivalent to NVQ level 4 or 5	KS13
socdeper	% persons aged >15 in social grades D or E	UV050
socdehh	% households in social grades D or E	UV104
rented	% households rented	KS18
medage	median age of population in the area	KS02
flookfam	% females aged 16-74 and economically inactive: looking after home/family	KS09
popsingle	% Persons aged 16 and over: Single (never married)	KS04
hhmardep	% households married with dependent children	KS20
hhcohabdep	% households that are cohabiting with dependent children	KS20
hhlonedep	% households made up of a lone parent with dependent children	KS22

Sources: KS tables obtained from http://www.ninis.nisra.gov.uk/mapxtreme/DataCatalogue.asp?button=Census.

The approximated social grade is obtained from:
http://www.ninis.nisra.gov.uk/mapxtreme/viewdata/Census/CensusUV050.xls and
http://www.ninis.nisra.gov.uk/mapxtreme/viewdata/Census/CensusUV104.xls.

PERCATH	% Persons with community background: Catholic1	KS07b

Table A1*.2. Results of principal component analysis on the selected census variables

Component	Proportion of variance	Cumulative	Rotated Component	Proportion of variance	Cumulative
1	0.548	0.548	*RPC1*	0.344	0.344
2	0.147	0.695	*RPC2*	0.276	0.620
3	0.131	0.826	*RPC3*	0.187	0.806
4	0.084	0.910	*RPC4*	0.104	0.910

Table A1*.3. The loadings of census variables on rotated components

	RPC1	*RPC2*	*RPC3*	*RPC4*
popnoqual	0.514	0.024	-0.066	-0.044
popdeg	-0.561	0.099	0.128	-0.014
socdeper	0.278	0.309	0.041	0.029
socdehh	0.295	0.302	0.027	0.018
rented	0.088	0.422	0.150	-0.023
medage	0.000	0.182	-0.708	-0.117
flookfam	0.406	-0.142	0.288	-0.159
popsingle	-0.216	0.377	0.499	-0.209
hhmardep	0.088	-0.636	0.240	-0.051
hhcohabdep	-0.016	0.004	0.020	0.922
hhlonedep	0.171	0.157	0.252	0.247
